# SEOM clinical guideline for the management of malignant melanoma (2017)

**DOI:** 10.1007/s12094-017-1768-1

**Published:** 2017-11-07

**Authors:** A. Berrocal, A. Arance, V. E. Castellon, L. de la Cruz, E. Espinosa, M. G. Cao, J. L. G. Larriba, I. Márquez-Rodas, A. Soria, S. M. Algarra

**Affiliations:** 1 Servicio de Oncología Médica, Consorcio Hospital General Universitario de Valencia, Avda. Tres Cruces 2, 46014 Valencia, Spain; 2 Hospital Clinic I Provincial de Barcelona, Barcelona, Spain; 3 Hospital Torrecárdenas, Almería, Spain; 4 Complejo Hospitalario Regional Virgen Macaren, Seville, Spain; 5 Hospital Universitario la Paz, Madrid, Spain; 6 Hospital Universitario Quirón Dexeus, Barcelona, Spain; 7 Hospital Universitario Clínico San Carlos, Madrid, Spain; 8 Hospital General Universitario Gregorio Marañón, Madrid, Spain; 9 Hospital Universitario Ramón y Cajal, Madrid, Spain; 10 Clínica Universitaria de Navarra, Pamplona, Spain

**Keywords:** Melanoma, Metastatic, Adjuvant, Immunotherapy, B-RAF

## Abstract

All melanoma suspected patients must be confirmed histologically and resected. Sentinel node biopsy must be done when tumor is over 1 mm or if less with high-risk factors. Adjuvant therapy with interferon could be offered for patients with high-risk melanoma and in selected cases radiotherapy can be added. Metastatic melanoma treatment is guided by mutational BRAF status. BRAF wild type patients must receive anti-PD1 containing therapy and BRAF mutated patients BRAF/MEK inhibitors or anti-PD1 containing therapy. Up to 10 years follow up is reasonable for melanoma patients with dermatologic examinations and physical exams.

## Methodology

As most of the knowledge on the treatment for this disease has come in the last 5 years and from phase III clinical trials for the development of this guideline, the authors have reviewed all phase III trials regarding the main aspects of this guideline and also the main guidelines on this disease. Recommendation and evidence have been graded according to the guidelines development recommendations [1].

## Surgical management of melanoma

Excisional biopsy with a 2 mm lateral margin and deep subcutaneous margin is indicated for any suspicious lesion (Grade recommendation A; Level of Evidence 1a). Once the diagnosis is confirmed by the pathologist, definitive surgery is done to obtain wide margins. The deep margin should extend to the fascia (Grade recommendation B; Level of Evidence 2b), whereas lateral margins will be determined by Breslow thickness: 1 cm if Breslow up to 1 mm; 1–2 cm for Breslow 1–2 mm; and 2 cm if Breslow > 2 mm (Grade recommendation A; Level of Evidence 1a). Wider margins do not provide benefit regarding recurrence or melanoma-related death rates [2].

Sentinel lymph node biopsy is recommended in melanomas over 1 mm depth (Grade recommendation A; Level of Evidence 1a). It can also be considered for melanomas with Breslow 0.75–1 mm of Breslow and any risk factor such as ulceration, Clark level IV, regression, increased mitotic rate or age less than 40 (Grade recommendation B; Level of Evidence 1a) [3]. Complete lymph node dissection of the involved nodal basin must be performed if sentinel node is positive or there are clinically positive nodes (stages IIB or IIIC) (Grade recommendation A; Level of Evidence 2a).

Surgical excision of solitary metastases is indicated whenever possible. Data from retrospective studies demonstrated survival rates of 20–30% at 5 years after surgical removal of single metastases (Grade recommendation B; Level of Evidence 2b).

## Adjuvant therapy

There is high risk of relapse for patients with stages IIB-C (T4 or with ulceration) and stage III (N positive). High-risk patients are considered candidates for adjuvant treatment. Interferon alpha high dose scheme (Induction treatment with 20 MU/m^2^ iv × 5 days/week × 4 weeks, followed by maintenance treatment with 10 MU/m^2^ sc × 3 days/week × 11 months) demonstrated a significant benefit in relapse-free survival versus observation. Although initially this benefit extended to overall survival, a follow-up superior to 12 years showed no significant differences [4]. After that, many studies have evaluated the efficacy and toxicity profile of this drug relative to other agents or different schemes and dosage. Low-dose interferon significantly improves RFS for stages II, but not significant in overall survival. However, evaluated in the global context of high-risk population (stages II and III) they are clearly inferior to high doses [5]. With all of these conflicting results about benefit in OS, recently several meta-analyses have tried to answer this question. Whereas one of them confirmed the significant improvement in RFS, but not for OS [6], other meta-analyses have demonstrated a significant benefit in OS [7]. Nevertheless, none of them have been able to respond the answer about the optimal IFNa treatment scheme and which subgroup of patients will be the best candidates to receive it.

Given these results, high-risk melanoma patients could be offered interferon adjuvant therapy unless there is a better treatment (Grade recommendation A; Level of Evidence 1a).

Adjuvant Ipilimumab at 10 mg/kg schedule has demonstrated in a phase III clinical trial (EORTC 18,071) an improvement in RFS and OS compared with placebo in resected stage III melanoma. More than 50% of patients experienced grade 3–4 adverse events, with a discontinuation rate of 32% in patients treated with ipilimumab, including 5 toxic deaths [8]. This indication is not approved in Europe, therefore no recommendation can be made.

A change is expected in the therapeutic scene in the next years with the publication of the results of trials evaluating new immunotherapy agents, such as nivolumab or pembrolizumab, and BRAF/MEK inhibitors.

## Radiotherapy

Adjuvant radiotherapy is rarely necessary for excised local melanoma and can be considered in the case of inadequate resection margins in lentigo maligno, desmoplastic neurotropic melanommma and also in the case of R1 resections of metastases when wide margins cannot be obtained (Grade of recommendation B; level of evidence 2b).

Adjuvant radiotherapy improves lymph-node field control with no effect on OS or RFS in patients at high risk of lymph node relapse following a lymphadenectomy for regional node involvement [9]. This strategy may be considered in selected patients with clinically appreciable nodes and features of high risk of nodal relapse such as extranodal tumor extension, ≥ 3 lymph nodes involvement and/or size of nodal metastasis ≥ 3 cm (Grade of recommendation C; level of evidence 1b). Its benefits should be weighed against potential long-term skin and regional toxicities.

Radiotherapy is an alternative for patients with in-transit metastases (Grade of recommendation C; level of evidence 4).

## Locorregional metastases

Patients with satellite and in-transit metastases should be treated within the context of clinical studies if possible.

Surgical therapy of in-transit metastases shall be performed when there is a possibility of macroscopic and microscopic complete removal of the metastatic lesions (Grade of recommendation B, level of evidence 4).

Intralesional therapy with Talimogene Laherparepvec has shown improvement in overall survival and locorregional control in patients with in patients with injectable lesions and unresectable stage IIIb/c or Iva specially when used as first line therapy [10] (Grade of recommendation B, level of evidence 1b).

In the presence of multiple, inoperable, locorregional cutaneous/subcutaneous metastases on an extremity, regional chemotherapy as isolated limb perfusion comes into consideration (Grade of recommendation B, level of evidence 4). Other procedures such as intralesional injection of interleukin-2 (Grade of recommendation B, level of evidence 4) or radiation therapy, electrochemotherapy, cryosurgery, or laser destruction may also be applied for local tumor control (Grade of recommendation C, level of evidence 4). Fundamental superiority of one over the other has not been proven and their use depends on individuals factors.

## Treatment of metastatic disease

All tumours should be tested for BRAF-V600 mutation (Grade of recommendation A, level of evidence 1a). Validated BRAF-V600E/K test methods are tissue based and provide qualitative data (positive or negative). Participation in clinical trials is strongly encouraged.

### First line therapy in BRAF-mutant, advanced (unresectable stages IIIC/IV) melanoma

The standard of care in this setting is the combination of a BRAF inhibitor and a MEK inhibitor (Grade of recommendation A, level of evidence 1a). Three phase III trials have demonstrated that combined therapy is more active than single agent BRAF inhibitor [11–13]. Over two-thirds of patients achieve an objective response, time to progression lies in the range of 10–12 months and median overall survival is around 25 months. The combination of encorafenib and binimetinib has also shown increased progression-free survival with regard to vemurafenib alone in an ongoing phase III study.

Single agent BRAF inhibitors (vemurafenib or dabrafenib) can be considered (response rate 50%, time to progression 6–8 months, median overall survival 16–18 months) if the combination with their companion MEK inhibitor cannot be used [14] (Grade of recommendation A, level of evidence 1a).

BRAF inhibitors are also active in patients with brain metastases: response rate 30–39%, overall survival 8 months [15]. Concomitant use with radiotherapy is not recommended due to the risk of increased toxicity (Grade of recommendation A, level of evidence 2a). The combination of BRAF and MEK inhibitors is currently being tested in this setting.

The anti-PD1 antibodies nivolumab and pembrolizumab are active in BRAF-mutant disease, although results seem to be inferior to those obtained in the BRAF-wild type population. Data come from phase III studies comparing pembrolizumab vs. Ipilimumab [16] and nivolumab vs. nivolumab plus ipilimumab vs. ipilimumab [17]. Anti-PD1 therapy can be considered in patients with good performance status who do not need a rapid response (Grade of recommendation B, level of evidence 2a).

There is limited experience with ipilimumab in this setting but as chemotherapy is a suboptimal treatment for first line in this population and should not considered as first line treatment.

### Second line therapy in BRAF-mutant advanced melanoma

If patient received immunotherapy as first line, the activity of BRAF and MEK inhibitors after immunotherapy has not been clearly prospectively studied, but seems to be similar to that obtained in first line in terms of response rate [18] (Grade of recommendation A, level of evidence 2b). Data from COLUMBUS trial (encorafenib + binimetinib), where BRAF mutant patients could have been treated previously with immune therapy, showed that these patients still benefit from the combination versus monotherapy (Grade of recommendation A, level of evidence 2a).

If the patient received BRAF/MEK inhibitors as first line therapy, the anti-PD1 antibodies nivolumab and pembrolizumab are superior to chemotherapy in second line. Data are limited in patients previously treated with BRAF inhibitors, but nivolumab and pembrolizumab yield more responses than chemotherapy in patients who have also received ipilimumab [19] (Grade of recommendation A, level of evidence 2a). Ipilimumab can be considered after anti PD-1 failure. Finally chemotherapy can be considered for patients who have exhausted other options (Grade of recommendation D, level of evidence 2b).

### Treatment of BRAF wild type, advanced (IIIC/IV) melanoma

Randomized trials have demonstrated the superiority of nivolumab and pembrolizumab in monotherapy in terms of response, PFS and OS compared to chemotherapy in untreated advanced melanoma patients [20] or Ipilimumab in first and second line treatment [16], with a favourable toxicity profile. Survival rates at 2 years were around 50%, and response rates of 44–37% for both anti-PD-1 inhibitors. These treatments must be considered as the first line treatment (Grade of recommendation A, level of evidence 1a).

In addition, the combination of nivolumab and ipilimumab and nivolumab monotherapy improved responses and PFS in patients with untreated advanced melanoma compared to ipilimumab [21]. Response rates are higher than with anti PD-1 alone, but also the toxicity that reaches 58.5% of grades 3–4. Nivolumab and Ipilimumab combination could also be considered as first line treatment but we can’t recommend witch population if any has better outcome (Grade of recommendation A, level of evidence 1a). The optimal duration of therapy, long-term results with these agents or criteria for the selection of patients for monotherapy vs combination immunotherapy are not yet known.

The value of Ipilimumab after anti PD-1 theray has not been assessed in clinical trials. Some reports on the patients that progressed in the first line trial with anti PD-1 therapies suggest that Ipilimumab maintains its response rate as second line and may add increased survival for patients progressing Anti PD-1(Grade of recommendation C Level of evidence 2b).

Several chemotherapeutic agents (dacarbazine, temozolamide, fotemustine, carboplatin, cisplatin, and paclitaxel among others) have been tested in randomized clinical trials with similar response rates (5–12%) and suvival (< 5%) results and should not be offered unless other treatment options are exhausted.

In patients with brain metastases, immunotherapy with ipilimumab or anti-PD1 antibodies have demonstrated some degree of activity, although evidence is extremely scarce up to now. In this situation, locoregional approaches (surgical resection, stereotactic radiosurgery and/or whole brain radiation) must be taken into consideration upfront. If the previous ones are discarded, then systemic therapies may be introduced judiciously [22].

Results from case reports and a phase II study of KIT inhibitor imatinib suggest that some patients with KIT mutations (more common in acral lentiginous and mucosal melanomas) may respond (10–20%) to KIT kinase inhibitor therapy (Grade of recommendation C, level of evidence 2b), but these agents have not been approved for this indication KIT as a therapeutic target in metastatic melanoma [23]. Same can be said for MEK inhibitors such as binimetinib in NRAS mutant melanoma, which have shown a superior PFS in comparison with DTIC (2.8 vs. 1.5 m), but with no effect on OS [24]. This indication is under evaluation, therefore no recommendation can be made.

## Follow up

The objective of follow-up is the early detection of recurrences and secondary melanomas. The optimal duration of follow-up remains controversial. Studies in stage I–III showed that 47% of recurrences occurred within the first year after diagnosis and 32 and 80% within the second and third years, respectively, and thorough follow-up is advocated for this time period. Late recurrences are well documented, but only 5% of recurrences occur after 10 years. Thus, a 10-year follow-up appears to be reasonable (Grade of recommendation B; level of evidence 1b). Patients with a primary melanoma are at increased risk for developing a second primary melanoma. Estimates of that increased risk range from 8 to 10%. Although the most secondary melanomas occur within the first two years after the primary diagnosis of melanoma may even occur more than 30 years after, suggesting a need for life-long, regular dermatologic examinations (Grade of recommendation B; level of evidence 3b). Self-examinations by the patient are an essential component of follow-up and can lead to early recognition of recurrences of new melanomas. The patients should receive instructions on self-examination to detect a new melanoma or recognize a recurrence themselves (Grade of recommendation B; level of evidence 3b).

Physical examinations in stage I–III disease have proven to be the most effective procedure for early recurrence detection [25] and shall be performed in all melanoma patients during follow-up (Grade of recommendation A; level of evidence 2b).

Routine blood testing to detect recurrence is not recommended (Grade of recommendation D; level of evidence 4). Early detection of locoregional lymph node metastases is of particular significance. In a meta-analysis of 74 trials, lymph node sonography proved to be the most sensitive and most specific procedure for the detection of locoregional lymph node metastases and is the particular interest in patients with and equivocal lymph node physical exam, patients without sentinel lymph node biopsy (SLNB) or patients with a positive SLNB who did not undergo complete lymph node disection (Grade of recommendation A; level of evidence 1a).

Overall, a general recommendation about imaging procedure is not possible, because there are no studies assessing how the early detection of a recurrence could have an impact in the overall survival with the new treatments, as immunotherapy. In view of the current data, it is possible that an early detection of recurrence could have an impact in the response and evolution with the new treatments. Individual follow-up exams may be conducted in a risk-adapted fashion, trimonthly intervals in high risk of recurrence and in patients with decreasing risk, follow-up intervals may be extended from 6 to 12 months.

**Table:** 

Recommendations table		
Surgery		
All melanoma suspected lesion must be biopsied	A	1a
Surgical margins should be Breslow adapted	A	1a
Melanomas of more than 1 mm should undergo sentinel node biopsy	A	1a
Melanomas of 0.75 mm should undergo sentinel node biopsy if there are risk factors	B	1a
Lymph node resection should be performed if sentinel node is positive or clinically evident	A	2a
Solitary metastases must be surgically removed	B	2b
Adjuvant therapy		
High risk melanoma patients could receive interferon adjuvant therapy	B	1a
If surgical margins are affected adjuvant radiotherapy may be added	B	2b
Adjuvant radiotherapy should be considered if more than 3 nodes are present, one is larger than 3 cm or capsule is broken	C	1b
Locoregional disease		
Palliative radiotherapy can be used in in transit metastases	C	4
Surgery can be used for in transit metastases	C	4
Isolated limb perfusion can be used for in transit metastases	C	4
T-VEC can be used in locorregional disease	B	1a
Metastatic disease		
B-RAF determination should be done for all metastatic patients	A	1a
Combined B-RAF/MEK inhibition should be offered for BRAF mutated patients	A	1a
Anti-PD1 containing therapy is the first option for BRAF wild type patients	A	1a
BRAF inhibitors may be used in brain metastases	A	2a
Anti PD1 based therapy can be an alternative for BRAF mutated patients whose disease is not aggressively progressing	B	2a
Chemotherapy is an option if no other therapy could be available	A	1A
Patients treated with immunotherapy must be offered BRAF/MEK therapy as second line	A	2b
Patients treated with BRAF/MEK inhibitors must be offered anti-PD1 based therapy	A	2a
KIT mutated melanomas may be offered KIT kinase inhibitors	C	2b
NRAS mutated melanomas may be offered encorafenib	C	2b
Follow up		
Ten year follow up must be offered	B	1b
Lifelong skin examination is recommended	B	3b
Self-examination is recommended	B	3b
Physical examination is recommended	A	2b
Lymph node sonogram is recommended if physical exam is not clear	A	1A
